# Quantifying Stress and Relaxation: The New Measure of Heart Rate Variability as a Precise Biomarker Beyond Beats

**DOI:** 10.1192/j.eurpsy.2025.2342

**Published:** 2025-08-26

**Authors:** I. Szendi, E. Rudics, A. Buzas, V. Bilicki, J. Dombi, A. Nagy, Z. Szabo, A. Palfi, A. Der

**Affiliations:** 1 Department of Psychiatry, Kiskunhalas Semmelweis University Teaching Hospital, Kiskunhalas; 2 Institute of Psychology; 3 Centre of Excellence for Interdisciplinary Research, Development and Innovation; 4 Doctoral School of Interdisciplinary Medicine, University of Szeged; 5 Institute of Biophysics, HUN-REN Biological Research Centre; 6 Department of Software Engineering; 7 Department of Computer Algorithms and Artificial Intelligence, University of Szeged, Szeged, Hungary

## Abstract

**Introduction:**

The basis of this study is established on the unreliability of conventional heart rate variability metrics. The complex physiology of stress and the relaxed state cannot be drawn sufficiently, because the conventional heart rate variability metrics, presented by the established professional medical literatures, are dependent on the changes in the heart rate and thus cannot provide reliable data.

**Objectives:**

We introduced a novel, heart rate independent variability parameter, which is the normalized variability, and we contrasted it with the consensus-based parameters (RMSSD: root mean square of successive differences between normal heartbeats; SDNN: standard deviation of normal-to-normal interbeat intervals).

**Methods:**

We tested the normalized variability parameter in two studies. During the first study, the work-related stress among professionals in the frontlines of healthcare was reduced during the work-shift via either heart rate variability-biofeedback training or through achieving a relaxed state by allowing the subjects of the test during breaks to relax in their own usual manner. Consequently, the sample of Study 1 was categorized into the heart rate variability biofeedback group (N = 21) and the habitual recreation group (N = 21). Comparatively, Study 2 was concluded on healthy students, where the subject sample consisted of N = 9 participants. In this case, stress response was triggered by one of two laboratory stress induction methods. This meant the application of either the Socially Evaluated Cold Pressor Test (SECPT) or, a novel stress induction procedure, the Socially Evaluated Stroop Test (SEST). Furthermore, we used the Kolmogorov-Smirnov test to compare the distribution of heart rate variability parameters, mean heart rate, logRMSSD, logSDNN, and normalized variability before, during, and after the stress-inducing and the stress-alleviating interventions.

**Results:**

According to our results, on the one hand, logRMSSD and logSDNN did not change significantly throughout the stress alleviation and stress inducing states; on the other hand, the distribution of normalized variability significantly changed during and after both stress decreasing methods (p ≤ 0.01) and between the period that preceded recreation and during the process of habitual recreation itself (p = 0.03). Normalized variability during and after the SECPT (p = 0.05) significantly changed as well; however, the heart rate did not change significantly under and during the test.

**Conclusions:**

Normalized variability, a heart rate variability parameter that is independent of the heart rate of the patient, can be considered a sensitive stress indicator and suitable for investigating the complexity of the functions of the vegetative nervous system without the confounding effect of the heart rate.

**Disclosure of Interest:**

None Declared

